# Ameliorative Mechanisms of ZnO Nanoparticles in Salinity-Stressed Basil Callus: From Redox Defense to Metabolomic Normalization

**DOI:** 10.3390/plants15203196

**Published:** 2026-10-18

**Authors:** Dilruba Tabay Sümbül, Damla Turan Büyükdinç, Aysel Özcan Aykutlu

**Affiliations:** 1 Central Research and Application Laboratories, Ağrı İbrahim Çeçen University, 04100 Agrı, Türkiye; dtabay@agri.edu.tr; 2 Department of Horticulture, Faculty of Agriculture, Recep Tayyip Erdoğan University, 53300 Rize, Türkiye; 3 Department of Field Crops, Faculty of Agriculture, Recep Tayyip Erdoğan University, 53300 Rize, Türkiye

**Keywords:** *Ocimum basilicum* L., callus culture, zinc oxide nanoparticles, salinity stress, reactive oxygen species, antioxidant enzymes, GC-MS metabolomics, metabolic reprogramming

## Abstract

Salinity is a major abiotic stressor inducing osmotic imbalance, ion toxicity, and oxidative damage in plant cells. This study investigates the ameliorative effects of 100 mg L^−1^ zinc oxide nanoparticles (ZnO NPs) on *Ocimum basilicum* L. callus cultures under salinity stress (0–90 mM NaCl) through an integrated enzymatic and GC–MS metabolomic approach. Exposure to 90 mM NaCl significantly suppressed biomass and increased oxidative stress markers (H_2_O_2_ and MDA). Notably, ZnO NP supplementation exhibited a selective modulatory role, preferentially stimulating the ascorbate–glutathione cycle, with ascorbate peroxidase activity reaching a peak of 1.16 U mg^−1^ protein. While ZnO NPs alone caused a slight increase in oxidative stress indicators, they significantly mitigated severe stress by reducing H_2_O_2_ levels and stabilizing protein content. Metabolomic profiling revealed that salinity triggered a shift toward sesquiterpene hydrocarbons (79.71%), whereas ZnO NP application reprogrammed secondary metabolism toward ester compounds (40.50%) and oxygenated metabolites, effectively normalizing salt-perturbed metabolite pools. Principal component analysis explained 72.5% of total variation, clearly distinguishing nanoparticle-driven recovery from stress-driven damage. These findings demonstrate that ZnO NPs enhance stress tolerance by reinforcing metabolic bridges between antioxidant defense and biomass preservation, offering a robust strategy for nano-enabled resilience.

## 1. Introduction

Soil salinization is a major constraint on plant productivity because it causes osmotic stress, ion toxicity, and oxidative stress, all of which can disrupt plant growth and metabolism. Reactive oxygen species (ROS), including hydrogen peroxide (H_2_O_2_), play an important role in plant responses to salinity. At controlled levels, ROS function as signaling molecules, whereas excessive accumulation can damage membranes, proteins, and nucleic acids. Plants therefore rely on efficient antioxidant defense systems to maintain cellular redox balance [1].

Basil (*Ocimum basilicum* L.), a plant native to tropical and subtropical climates, is an important medicinal and aromatic plant belonging to the Lamiaceae family that contains essential oils [2]. The presence of phytochemicals such as terpenes, phenylpropanoids, alcohols, and aldehydes in basil has led to its utilization in the food industry as a flavoring and fragrance agent. In traditional medicine, it has been employed in a variety of applications, including aromatherapy, as a carminative, urinary tract antiseptic, analgesic, expectorant, and cough suppressant, and for the treatment of bad breath and dental odors. It has also been utilized in the management of diarrhea, chronic dysentery, cardiovascular diseases, Alzheimer’s disease, and various cancers [3,4,5].

Purple basil cultivars, characterized by distinct pigmentation and specialized metabolism, provide an attractive platform for probing stress-linked metabolic plasticity. Controlled salinity has been shown to modulate basil morphometrics, mineral balance, and bioactive metabolome, underscoring the complex trade-off between biomass penalties and metabolite remodeling [6].

Nanotechnology-based approaches, particularly micronutrient-derived nanoparticles, have been proposed to enhance abiotic stress tolerance in plants. Zinc is an essential micronutrient involved in enzyme structure/function and redox homeostasis. ZnO nanoparticles have been reported to mitigate salt stress in multiple species by improving antioxidant capacity, photosynthetic performance, and ionic balance [7,8,9]. However, nanoparticle effects are dose- and context-dependent; inappropriate doses may induce phytotoxicity, highlighting the need for careful concentration selection and mechanistic evaluation [10,11]. The synthesis of specialized metabolites in basil can be increased as a component of the adaptive response of plants to abiotic stressors such as drought, salinity, UV radiation, and exposure to NPs [12,13,14].

Callus cultures offer a reductionist, highly controlled system to dissect stress responses with minimized whole-plant transport complexity. Because nanoparticles can influence metabolism via ROS bursts and downstream signaling, integrated biochemical and metabolomic readouts are particularly informative [15]. Metabolomics has been emphasized as a key tool for priming research because it captures coordinated reprogramming of metabolite pools underlying stress acclimation [16].

In this study, we tested the hypothesis that ZnO nanoparticles mitigate salinity stress in purple basil callus cultures by (i) reinforcing enzymatic antioxidant defenses to reduce ROS-driven lipid peroxidation and (ii) reshaping salinity-induced metabolic reprogramming detectable by GC-MS. By integrating oxidative stress markers, antioxidant enzyme activities, and untargeted metabolomics, we aimed to build a system-level view of nano-enabled salinity mitigation in an in vitro basil platform.

## 2. Results

### 2.1. Callus Growth Parameters Under Salinity

**Figure 1:**
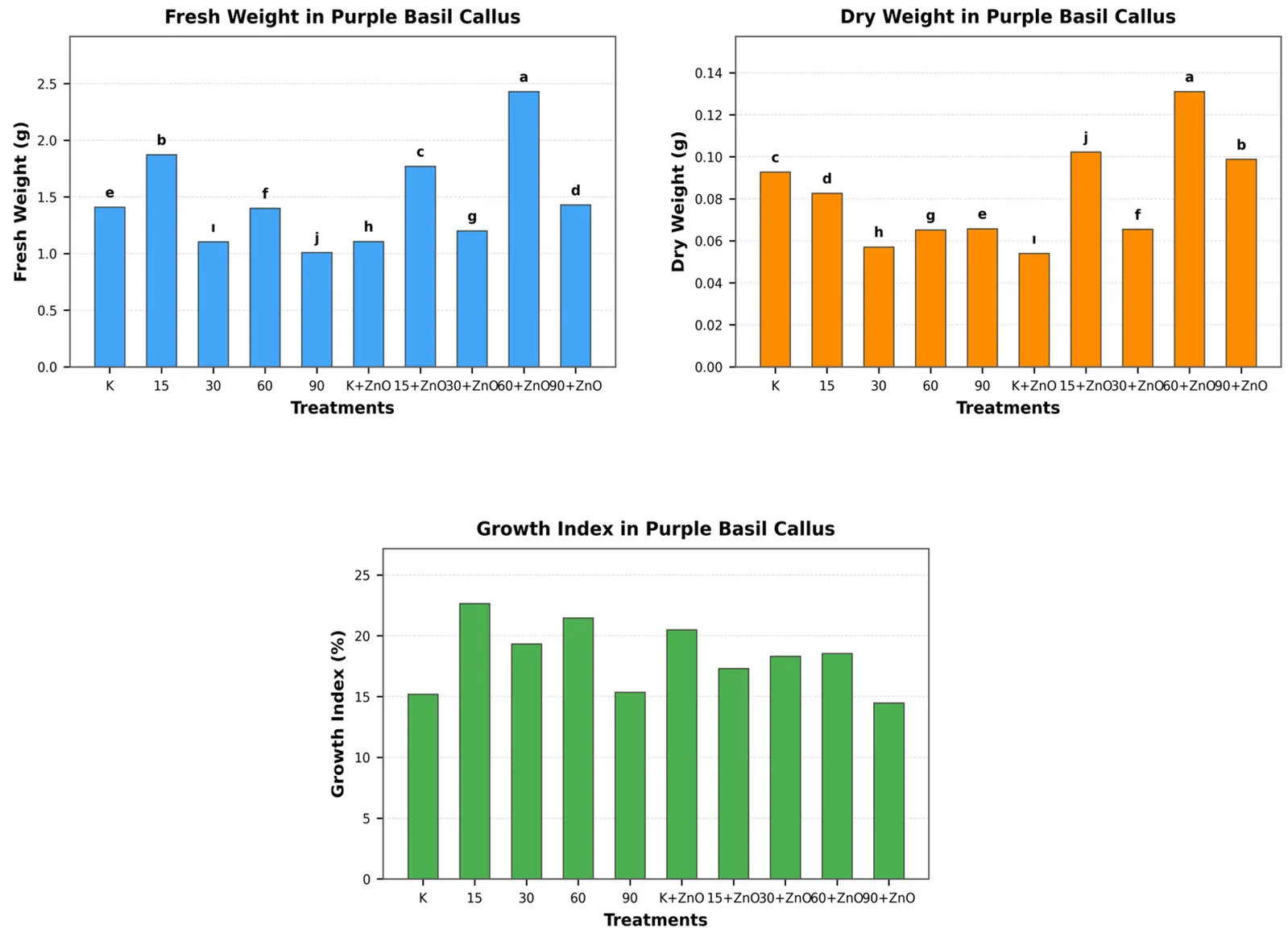
Growth traits of purple basil callus under salinity and ZnO NP treatments. Values with different lowercase letters within the same column are significantly different according to Tukey’s multiple range test (p<0.05).

The effects of salinity and ZnO nanoparticle applications on callus growth in purple basil (*Ocimum basilicum* L. cv.‘Red Rubin’) are presented in Figure 1.

Fresh weight, dry weight, and growth index values clearly varied depending on both salt concentration and ZnO NP treatment. Moderate salinity levels (15 and 60 mM) resulted in higher growth index values compared to the control, suggesting that low-to-moderate stress may stimulate callus growth to some extent. In contrast, high salinity (90 mM) had a negative effect on growth, leading to reduced values.

ZnO nanoparticle application supported callus development, especially under saline conditions. The highest fresh and dry weight values were obtained in the 60 + ZNP treatment, indicating a positive effect of ZnO at moderate salinity levels. The relatively stable growth index values in ZnO NP-treated groups also suggest that ZnO helps maintain growth under stress conditions. Overall, these findings indicate that ZnO nanoparticles can alleviate the negative effects of salinity and support callus growth, particularly at moderate stress levels.

### 2.2. ZnO NPs Differentially Modulated Salinity-Induced Oxidative Damage

**Figure 2:**
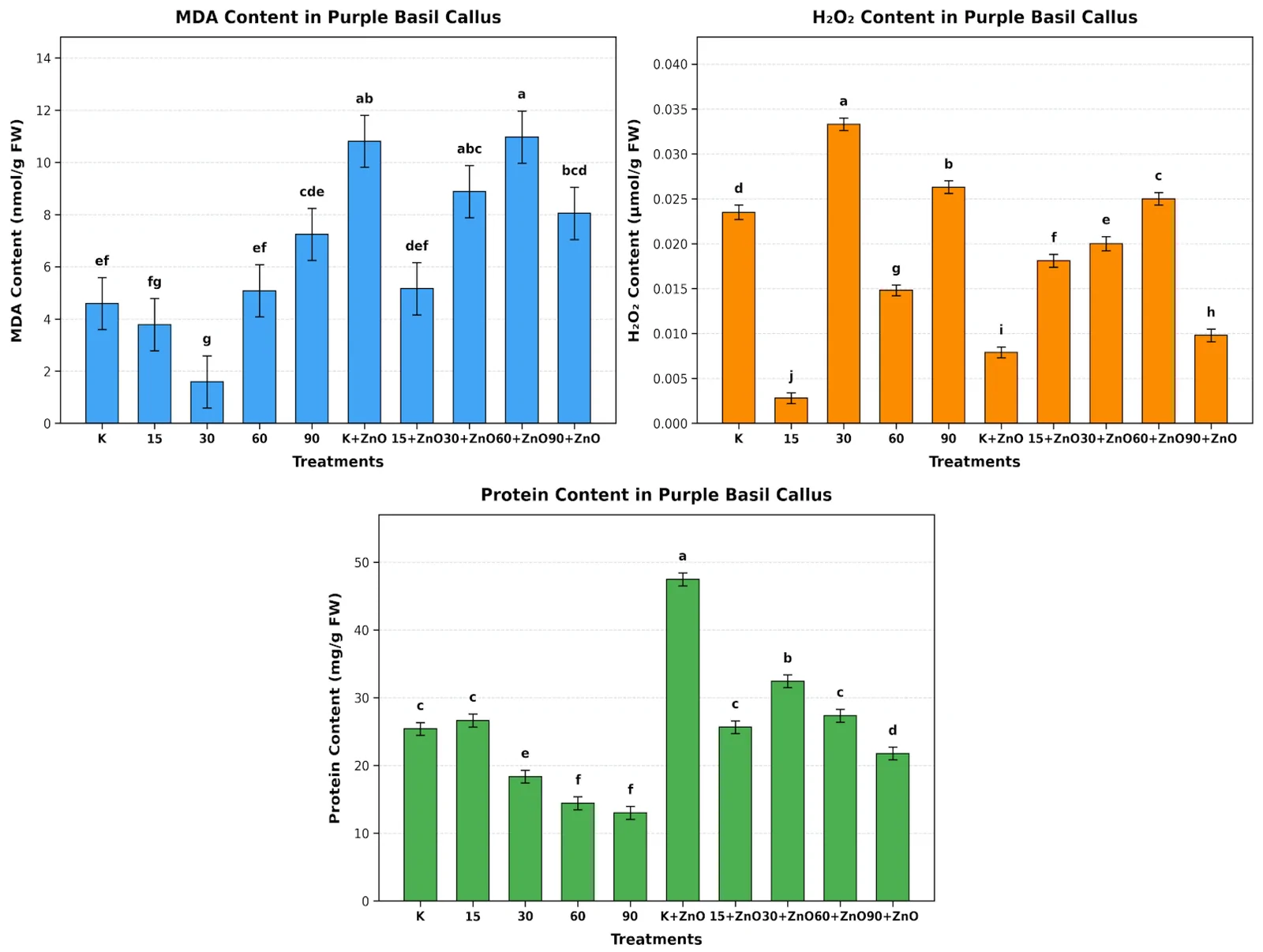
Physiological and biochemical responses of purple basil (*Ocimum basilicum* L.) callus to different treatment combinations: MDA content (nmol/g FW), H_2_O_2_ content (µmol/g FW), and total protein content (mg/g FW). Bars represent mean ± SD (n=3). Values with different lowercase letters above the bars indicate statistically significant differences according to Tukey’s HSD test (p<0.05).

The impact of various treatment concentrations with and without zinc supplementation on MDA, $H_{2}O_{2}$, and protein accumulation in purple basil callus is illustrated in Figure 2. Soluble protein content in basil callus tissues exhibited a significant decline with increasing salt concentrations; the protein level decreased from 25.41 mg/g in the control group to 13.01 mg/g under 90 mM NaCl treatment. In contrast, the application of 100 mg L^−1^ ZnO nanoparticles (ZnO NPs) alone resulted in the highest protein content, increasing it approximately twofold (47.46 mg/g). Combined treatments of ZnO NPs with salinity markedly enhanced protein levels compared to salt-only treatments across most salinity levels (e.g., from 18.36 to 32.44 at 30 mM), suggesting that ZnO nanoparticles may support protein synthesis and/or enhance protein stability.

Analysis of H_2_O_2_ levels revealed that low salinity (15 mM) reduced hydrogen peroxide accumulation, whereas higher concentrations (≥30 mM) led to fluctuating changes. ZnO NP application decreased H_2_O_2_ levels, particularly under high salinity (90 mM). However, the increase in H_2_O_2_ observed at some moderate salinity levels shows that the response to ZnO NPs was not consistent across all treatments. Their effects appeared to depend on the severity of salt stress, with beneficial effects becoming more evident under more severe stress conditions.

MDA increased with rising salinity, particularly at 60 and 90 mM NaCl. ZnO NP application alone resulted in the highest MDA level (10.81 nmol g^−1^ FW), indicating increased lipid peroxidation in the absence of salt stress. In contrast, ZnO NP treatment reduced MDA accumulation under high salinity (90 mM), suggesting that its effect on oxidative damage may vary depending on the severity of salt stress.

This dual response indicates that the effect of ZnO NPs on oxidative stress varied with salinity level, as reflected by changes in H_2_O_2_ and MDA levels.

### 2.3. ZnO NPs Differentially Regulated Enzymatic Antioxidant Defenses Under Salinity

**Figure 3:**
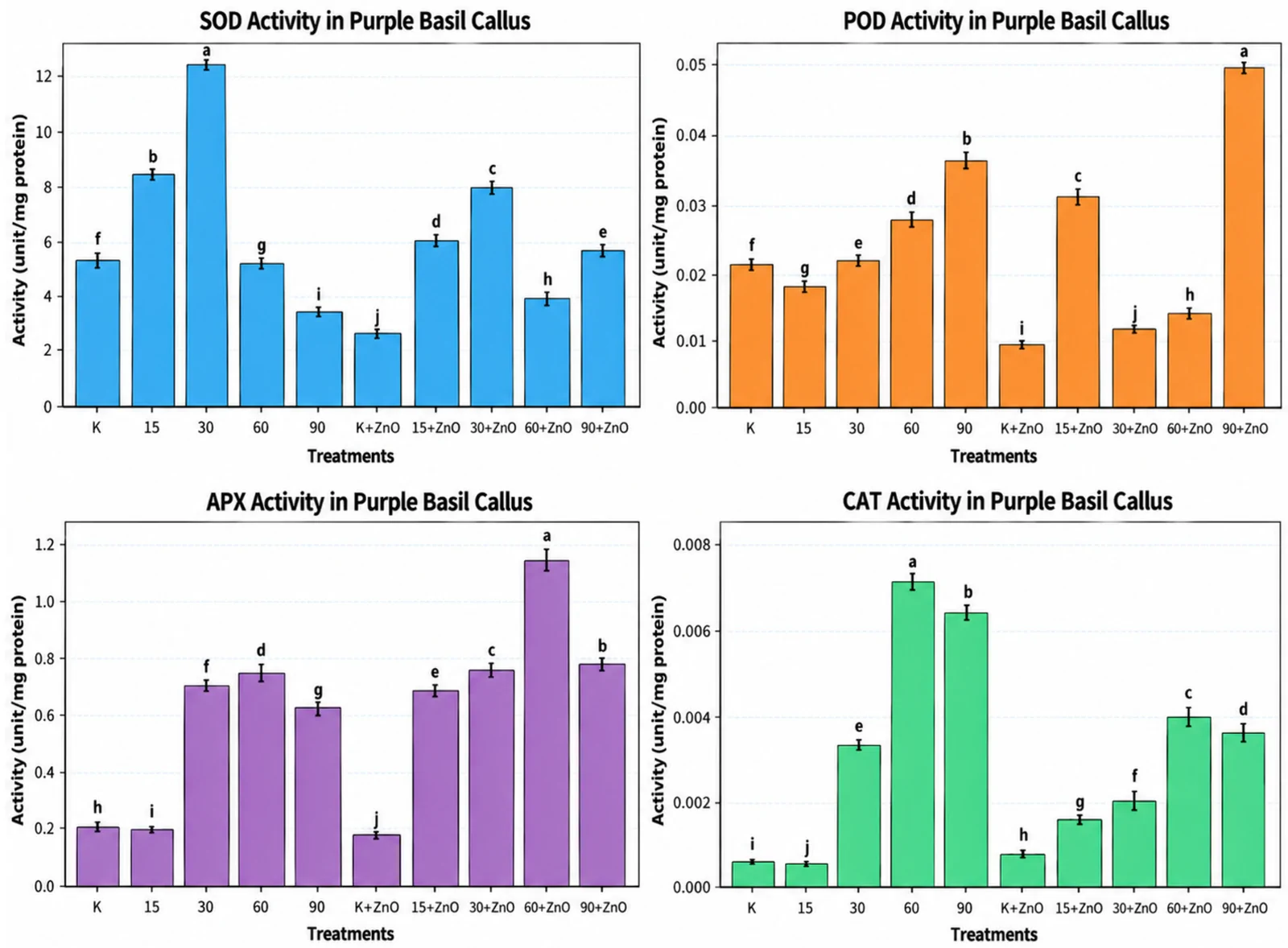
Antioxidant enzyme activities of purple basil (*Ocimum basilicum* L.) callus in response to different treatment combinations: SOD (superoxide dismutase) activity (unit/mg protein), POD (peroxidase) activity (unit/mg protein), APX (ascorbate peroxidase) activity (unit/mg protein), and CAT (catalase) activity (unit/mg protein). Bars represent mean ± SD (n=3). Values with different lowercase letters above the bars indicate statistically significant differences according to Tukey’s HSD test (p<0.05). K: control.

Activities of key antioxidant enzymes (SOD, CAT, APX, POD) were significantly affected by salinity and ZnO NP treatment (Figure 3). Salinity triggered a stress response characterized by different amounts of NaCl. ZnO NP supplementation enhanced the activity of major ROS-scavenging enzymes under salinity, contributing to lower oxidative damage markers relative to salt-only calli.

SOD forms the first line of oxidative defense by converting superoxide radicals into hydrogen peroxide [17]. Our findings show that SOD activity increased markedly under moderate salinity (30 mM), reaching its peak, but declined at higher salt concentrations. While ZnO NP application reduced SOD activity under moderate stress, the subsequent increase under severe salinity (90 mM) is noteworthy. This pattern suggests that ZnO nanoparticles may trigger a compensatory response under severe stress conditions, thereby contributing to the detoxification of superoxide radicals.

Analysis of the data revealed that POD activity increased in response to salinity stress, reaching its maximum level at 90 mM NaCl. ZnO NP application enhanced POD activity in certain treatments (15 and 90 mM), indicating an improved antioxidant response. In contrast, the suppression of POD activity in the 30 and 60 mM salinity + ZnO NP combinations suggest a selective and concentration-dependent modulation of the enzymatic defense system by ZnO nanoparticles.

CAT activity showed a pronounced increase, particularly at 60 mM salinity, reflecting a strong response. ZnO NP treatment suppressed CAT activity under low and moderate salinity but exerted a stabilizing effect under high stress conditions. When evaluated together with H_2_O_2_ levels, these changes indicate that the antioxidant system operates in a dynamic and only partially coordinated manner, being reorganized depending on stress severity. These results indicate that ZnO NPs may enhance hydrogen peroxide scavenging mechanisms; however, this effect appears to be enzyme-specific and condition-dependent [17,18].

According to the analysis results, ZnO NP application significantly increased APX activity. The APX level, which was 0.734 U mg^−1^ protein at 60 mM salinity, reached 1.160 U mg^−1^ protein in the 60 mM + ZnO treatment group, representing the highest value among all treatments. These findings demonstrate that ZnO nanoparticles prominently stimulate APX activity under salinity stress, suggesting that the ascorbate–glutathione cycle plays a key compensatory role in enhancing cellular defense mechanisms, with ZnO NPs preferentially stimulating APX activity rather than uniformly enhancing all antioxidant enzymes.

### 2.4. Principal Component Analysis and Inter-Variable Relationships

**Figure 4:**
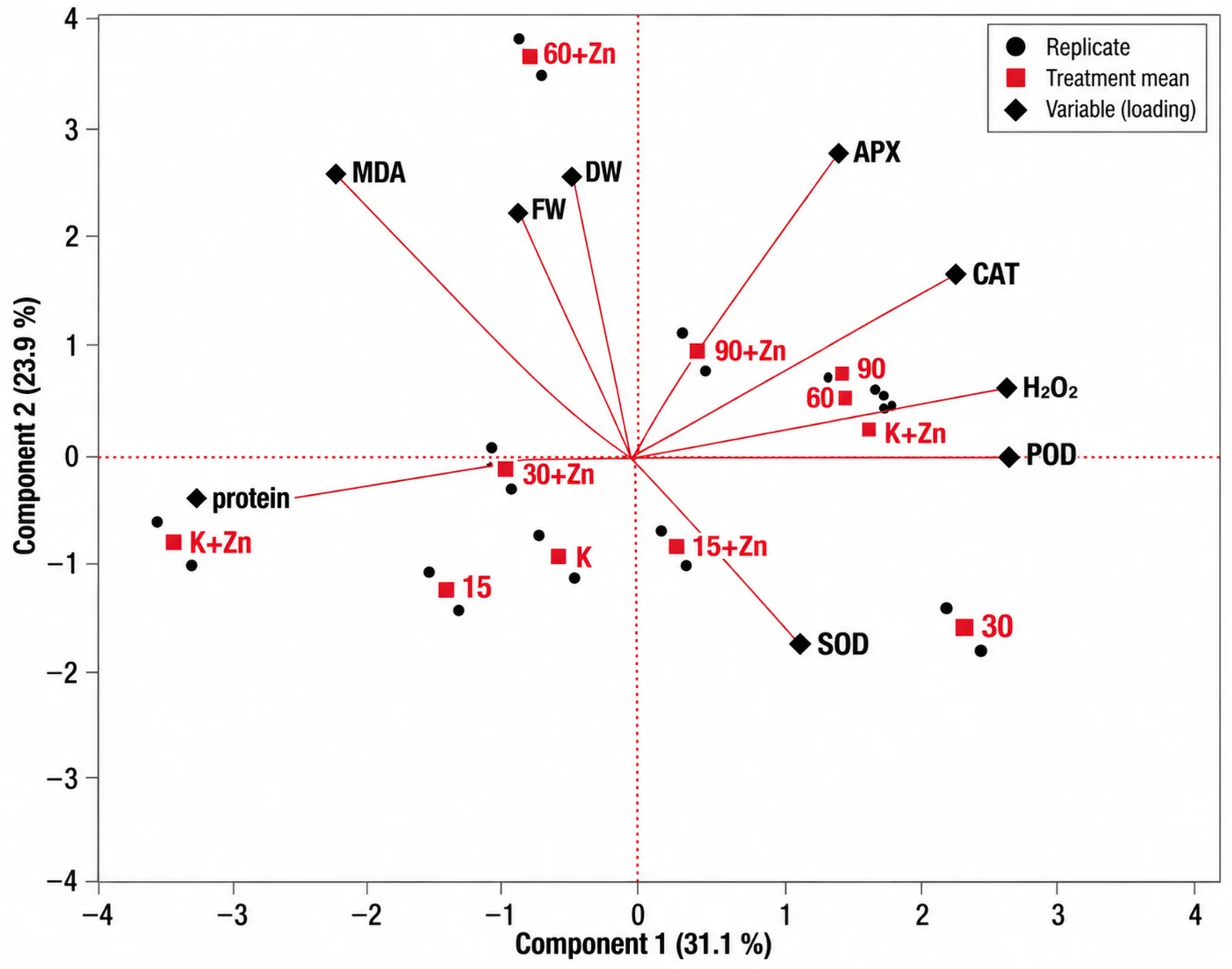
PCA biplot showing the relationships among physiological and biochemical parameters.

A principal component analysis (PCA) was conducted to evaluate the responses of callus tissues to salt stress and ZnO NP treatments from a holistic perspective (Figure 4). The analysis revealed distinct clustering patterns among treatment groups and measured parameters, indicating differential physiological responses. PCA further highlighted the complex metabolic reprogramming induced by salinity and ZnO NP application in purple basil callus cultures.

The protein vector is located in the negative region of PC1, while the POD, CAT, and APX vectors lie in the positive region. This opposite alignment indicates an inverse relationship between protein content and antioxidant enzyme activities. This pattern indicates that protein accumulation tends to decrease under stress conditions, whereas antioxidant defense mechanisms are activated. Additionally, the proximity of the MDA vector to fresh weight (FW) and dry weight (DW) along PC2 suggests a potential association between biomass parameters and oxidative stress indicators.

The distribution of treatment groups further illustrates dose-dependent effects. The K + Zn (control + ZnO NP) group clustered near the protein vector, indicating enhanced protein accumulation under non-stress conditions. In contrast, high salinity treatments (60, 90, 60 + Zn, and 90 + Zn) were located closer to APX, CAT, and POD vectors, suggesting increased antioxidant enzyme activity under severe stress. Notably, the proximity of the 60 + Zn group to the MDA vector corresponds with elevated lipid peroxidation levels observed in this treatment. Furthermore, the positioning of the 30 mM NaCl treatment near the SOD vector indicates that superoxide dismutase represents a primary defense response at moderate stress levels. Overall, PCA results suggest that ZnO nanoparticles contribute to a shift in metabolic responses under salinity stress, from growth- and protein-associated processes toward enhanced antioxidant defense and redox regulation. In particular, the association of 60 + Zn and 90 + Zn treatments with enzyme vectors indicates that ZnO NPs may facilitate stress adaptation through modulation of antioxidant pathways, especially involving APX and POD.

**Figure 5:**
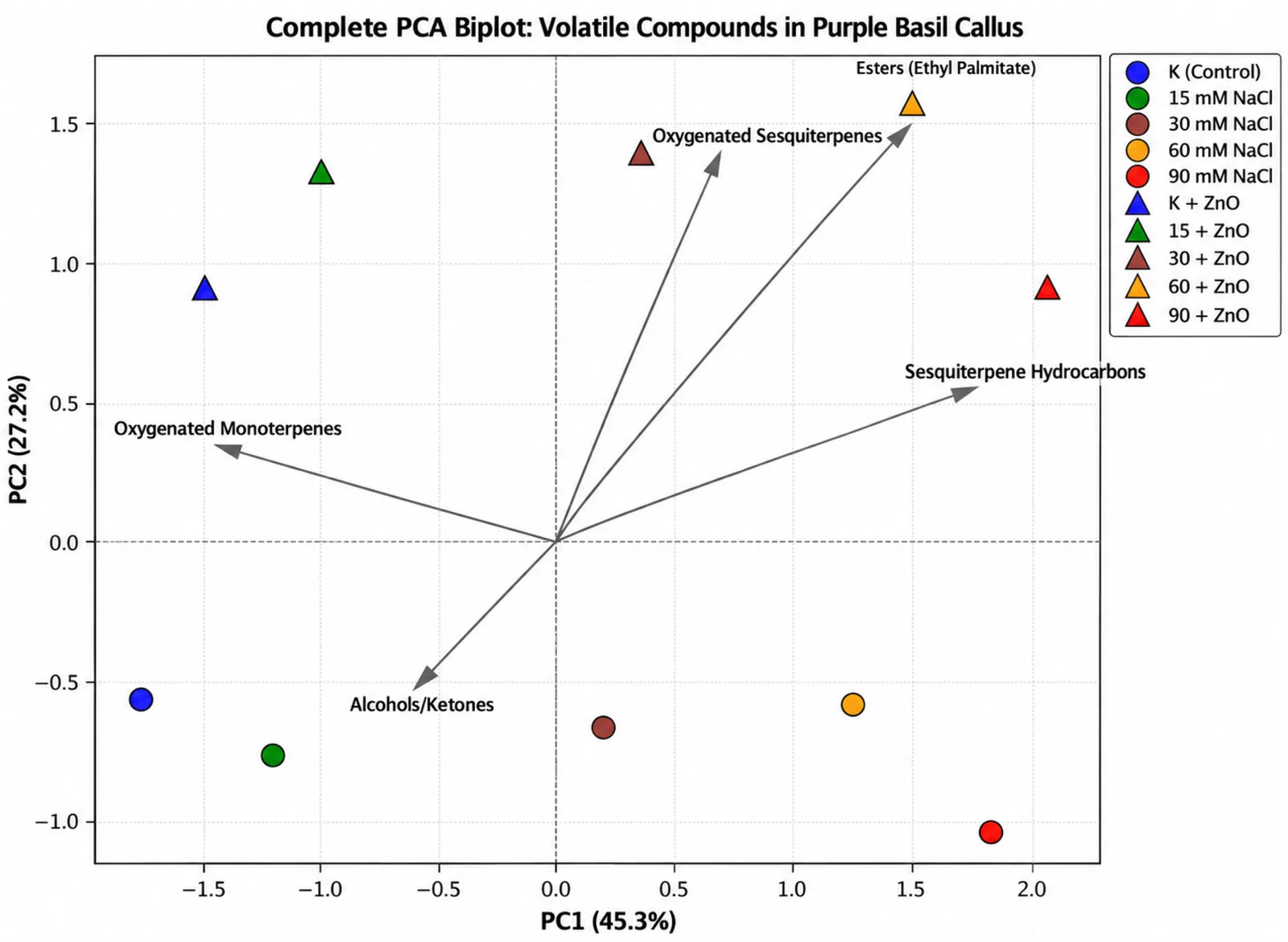
Distribution of purple basil callus samples based on their volatile compound profiles.

Principal component analysis (PCA) revealed distinct distribution patterns among purple basil callus samples treated with varying levels of NaCl and ZnO (Figure 5). The first two principal components explained 72.5% of the total variance, with PC1 accounting for 45.3% and PC2 for 27.2%. Samples from the control group (K) and lower-salinity treatments (15 and 30 mM NaCl) without or with low ZnO (15 + ZnO) were grouped on the negative PC1 axis, exhibiting a close association with the vector representing alcohols and ketones.

Conversely, treatments combining higher salinity with ZnO—particularly 60 + ZnO and 90 + ZnO—shifted strongly along the positive PC1 and PC2 axes. These high-stress treatments showed strong positive alignment with vectors corresponding to esters (specifically ethyl palmitate), sesquiterpene hydrocarbons, and oxygenated sesquiterpenes. Samples treated with intermediate NaCl concentrations and ZnO (e.g., 30 + ZnO) displayed moderate positioning along PC2, showing a separate alignment relative to oxygenated sesquiterpenes. Overall, oxygenated monoterpenes loaded predominantly toward the negative PC1 axis.

**Figure 6:**
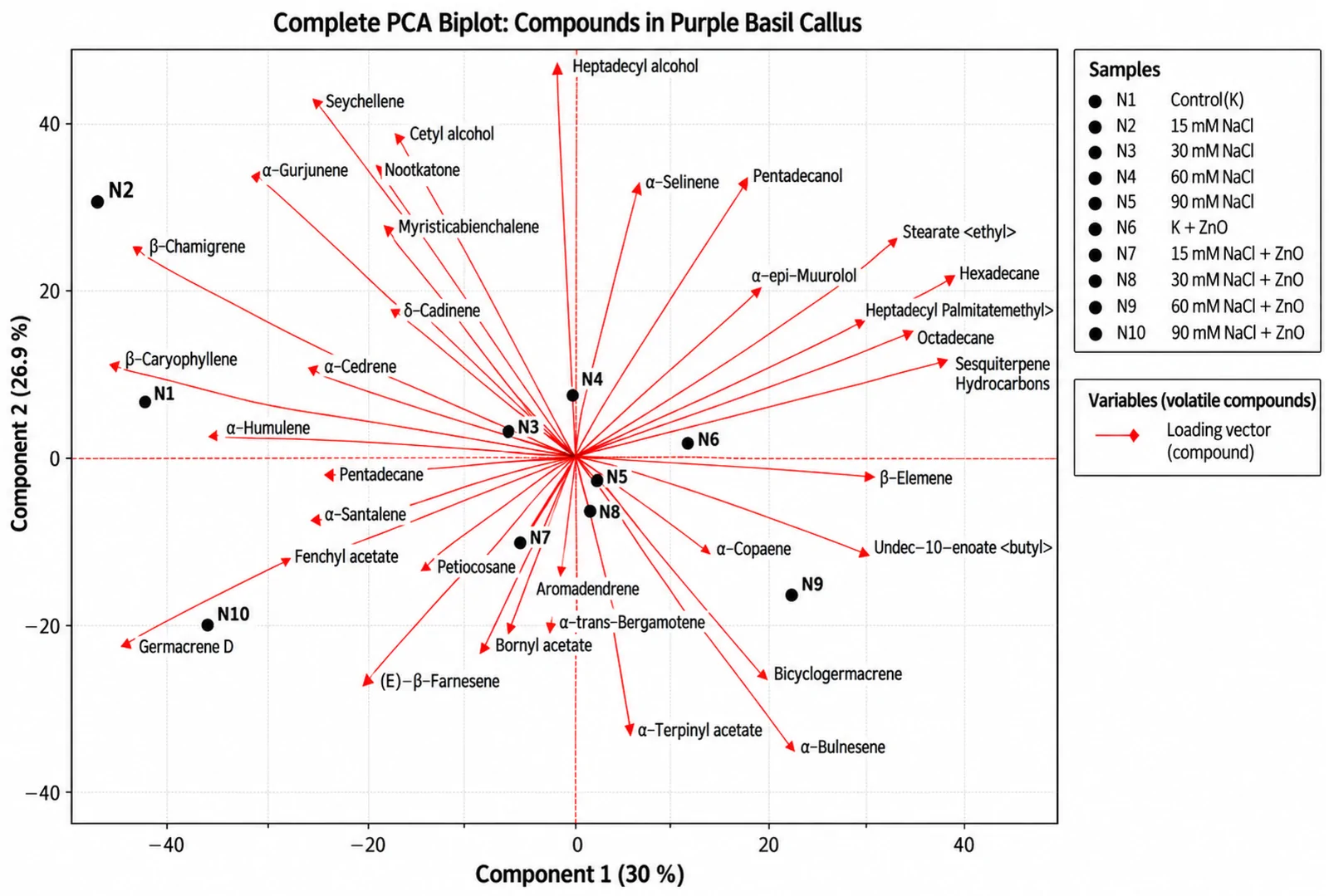
Principal component analysis (PCA) biplot of the analyzed samples and chemical compounds.

Building upon the general distribution of chemical classes, principal component analysis (PCA) based on individual volatile compounds further clarified the distinct grouping among the purple basil samples (PC1: 30.0%, PC2: 26.9%; Figure 6).

Most samples (N1–N4, N9, and N10) clustered tightly around the origin, reflecting a comparable baseline profile. In contrast, N5 and N6 shifted toward positive PC1, showing strong alignment with long-chain hydrocarbons and esters such as hexadecane, tetradecane, octadecane, methyl palmitate, and ethyl stearate. Sample N8 separated along negative PC2, closely associated with α-copaene. Notably, N7 stood out as a clear outlier in the lower-left quadrant (negative PC1 and PC2), driven by high relative contributions of sesquiterpene and ester vectors, particularly germacrene D, (E)-β-farnesene, fenchyl acetate, and α-santalene.

### 2.5. Correlation Heatmap and Statistical Interrelationships

**Figure 7:**
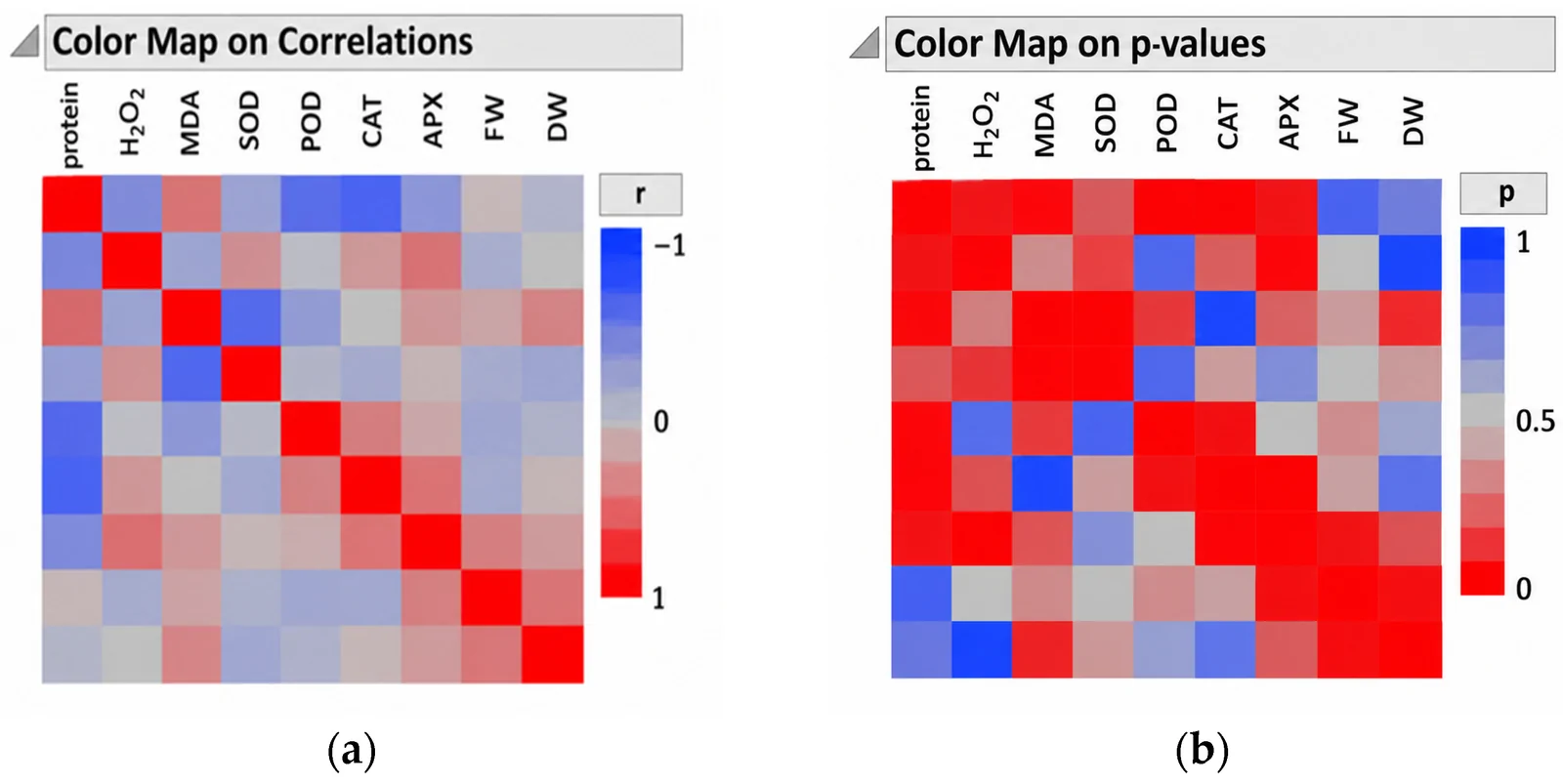
Pearson correlation heatmap (**a**) and *p*-value matrix (**b**) illustrating the interrelationships among growth traits (FW and DW), oxidative stress markers (H_2_O_2_ and MDA), and antioxidant enzyme activities (SOD, POD, CAT, and APX) in purple basil callus cultures. In the heatmap, red indicates a positive correlation (r → 1), blue indicates a negative correlation (r → −1), and grey indicates no correlation. In the *p*-value map, red indicates statistically significant relationships (*p* < 0.05).

To further elucidate the coordinated physiological and biochemical responses in purple basil callus, a Pearson correlation analysis was conducted, integrating all quantified parameters (Protein, H_2_O_2_, MDA, SOD, POD, CAT, APX, FW, and DW). The resulting correlation heatmap and *p*-value matrix (Figure 7) provide a comprehensive overview of the metabolic interconnectedness under salinity and ZnO NP treatments.

The correlation analysis revealed several highly significant (*p* < 0.05) relationships that define the stress-response architecture of the callus.

A strong positive correlation was observed among the activities of POD, CAT, and APX (indicated by intense red cells in the correlation map and significant *p*-values). This synergy confirms a highly coordinated enzymatic defense mechanism designed to neutralize H_2_O_2_ and mitigate oxidative damage. Protein content exhibited a strong negative correlation with H_2_O_2_ and MDA levels (blue cells in the correlation map). This indicates that the surge in reactive oxygen species and subsequent lipid peroxidation directly contributed to the degradation or inhibition of total protein accumulation. Fresh weight (FW) and dry weight (DW) showed a significant positive correlation with each other, as expected. Interestingly, they exhibited moderate to strong positive correlations with specific antioxidant enzymes (e.g., APX and POD), suggesting that the maintenance of biomass under stress is closely tied to the efficiency of the enzymatic scavenging system. SOD activity showed a distinct pattern, often exhibiting lower correlation coefficients with other enzymes like CAT or APX. This suggests that SOD operates as an independent first-line defense, primarily responding to the initial superoxide burst rather than the subsequent H_2_O_2_ detoxification pathway.

### 2.6. GC-MS Metabolomics Revealed Metabolic Reprogramming and ZnO-Enabled Recovery: Differential Metabolites and Pathway Enrichment

Salinity significantly altered the composition of volatile compounds identified in the callus tissues of *Ocimum basilicum* L. (purple basil). These metabolites were classified into major chemical groups, including oxygenated monoterpenes, sesquiterpene hydrocarbons, oxygenated sesquiterpenes, alcohols/ketones/aldehydes/furans, alkanes/alkenes, and esters (Table 1).

**Figure 8:**
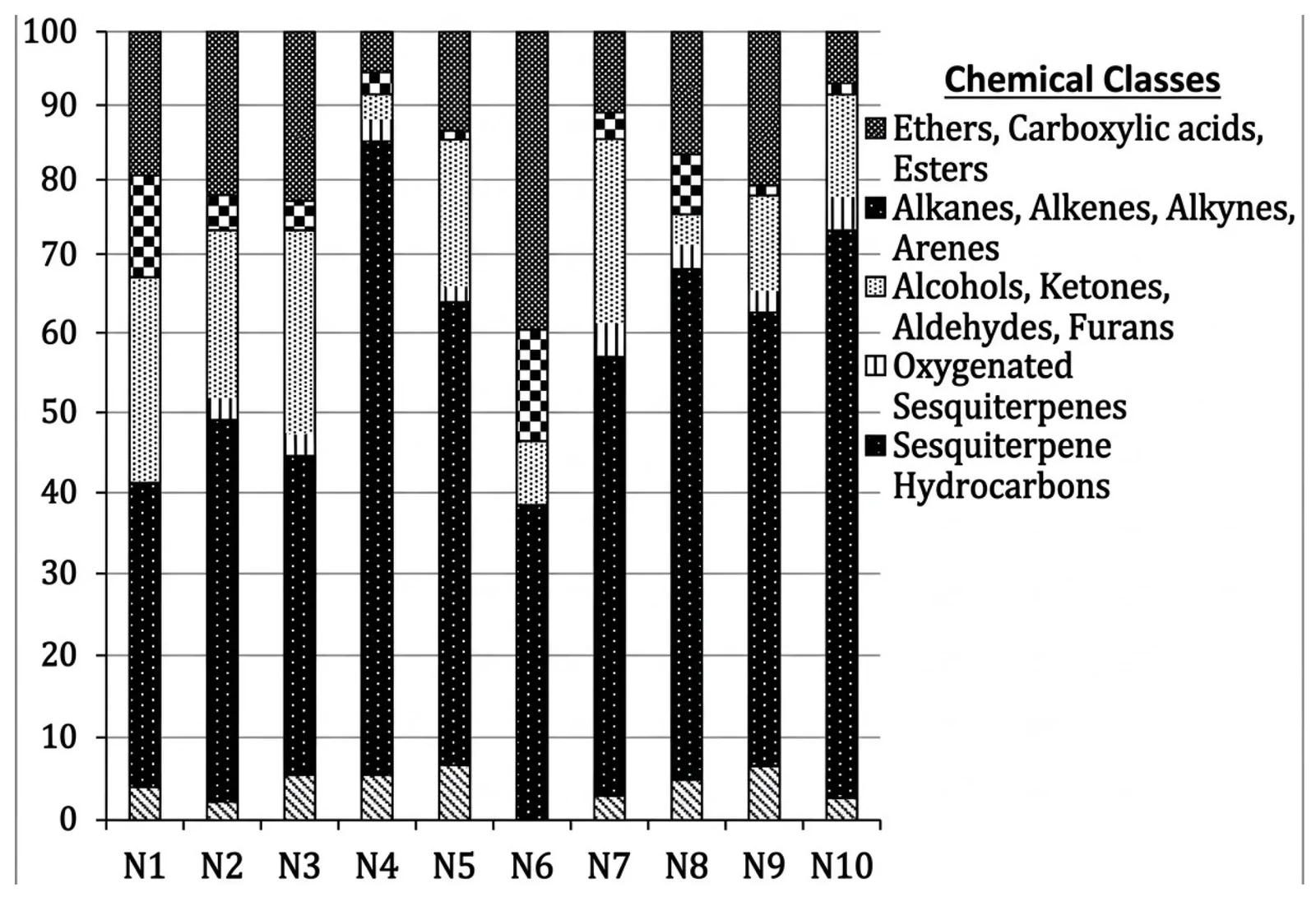
Relative chemical composition (%) of volatile profiles identified in samples N1 to N10 categorized by functional groups depending on salt and ZNP treatments.

Oxygenated monoterpenes were detected in varying proportions across treatments, with total content ranging between 2.09% and 6.49%. Sesquiterpene hydrocarbons represent the dominant fraction, with total percentages ranging from 37.11% to 79.71%, indicating their primary role in the volatile profile. Oxygenated sesquiterpenes’ total contribution was relatively low, ranging from ~2.33% to 4.40%. The total content of alcohols, ketones, aldehydes, and furans ranged between 3.49% and 26.28%, with pentadecanol and methyl eugenol being prominent compounds. Alkanes, alkenes, alkynes, and arenes were present in relatively lower proportions, ranging from 1.33% to 13.93%. Ethers, carboxylic acids, and esters showed considerable variation among treatments, with total values ranging from 6.15% to 40.50%, where ethyl palmitate was the dominant compound. Sesquiterpene hydrocarbons were the predominant class in all treatments, indicating their major role in stress response and metabolic regulation. In contrast, oxygenated monoterpenes and oxygenated sesquiterpenes were present in lower amounts but may play critical roles in signaling and adaptive responses (Figure 8). Variations in ester and long-chain hydrocarbon contents suggest that salinity and ZnO NP treatments significantly altered membrane-related and secondary metabolic pathways.

## 3. Discussion

In the present study, the effects of 100 mg L^−1^ ZnO nanoparticles (NPs) on purple basil callus exposed to salinity were examined using oxidative stress parameters together with GC–MS metabolomic analysis. Changes in H_2_O_2_ and MDA indicated that salinity affected the oxidative status of the callus tissues, in line with the well-established relationship between ROS accumulation and membrane damage under stress [1]. Interestingly, ZnO NPs alone increased protein content but also resulted in higher MDA levels. This suggests that, under non-stress conditions, ZnO NPs may stimulate metabolic activity while also imposing some degree of oxidative stress [17,18,19].

The antioxidant enzymes did not respond to ZnO NPs in the same way, and their responses varied with salinity level. APX activity increased with ZnO NP supplementation, particularly under moderate salinity, whereas CAT activity was lower under the same conditions. One possible explanation is the different roles of these enzymes in H_2_O_2_ removal. APX has a higher affinity for H_2_O_2_ and can function effectively at lower H_2_O_2_ concentrations than CAT [20]. Changes in Zn availability following ZnO NP application may also influence metal homeostasis and enzyme activity, although this was not directly examined in the present study [17]. SOD and POD also showed different responses across salinity levels, indicating that the effect of ZnO NPs on antioxidant defense depends on the severity of salt stress. Changes in ROS balance, Zn availability, and cellular redox status may be involved in these responses [21,22]. The correlation analysis was consistent with this overall pattern, showing coordinated changes among oxidative stress and antioxidant parameters rather than a general increase in all antioxidant enzymes.

The GC–MS results showed that salinity and ZnO NP supplementation also affected primary metabolism, including sugars, organic acids, and amino acids. Some metabolites showed patterns closer to the control following ZnO NP treatment, which may indicate partial metabolic adjustment under salinity [16,23,24]. Differences were also observed in the overall chemical profiles of the samples, consistent with the considerable chemical diversity previously reported in *Ocimum basilicum* L. and its cultivars and chemotypes [25,26,27,28]. PCA further showed that several chemical groups and individual compounds contributed to the separation among treatments [28,29].

Overall, the findings indicate that ZnO NPs altered antioxidant and metabolic responses in purple basil callus, but their effects varied with salinity level and were not uniformly protective. The mechanisms underlying these responses cannot be fully resolved from the present data. In particular, only one ZnO NP concentration (100 mg L^−1^) was tested, and nanoparticle behavior in the culture medium, dissolved Zn^2+^, metal accumulation, and antioxidant gene expression were not measured. Future studies addressing these factors and testing a wider range of ZnO NP concentrations would help clarify whether the observed responses are related to the nanoparticles themselves, released Zn ions, or a combination of both [10,11].

## 4. Materials and Methods

This study was conducted in 2025 at the Tissue Culture Laboratory, Faculty of Agriculture, Recep Tayyip Erdoğan University, Rize, and the Central Research and Application Laboratories, Ağrı İbrahim Çeçen University, Ağrı, Türkiye. Seeds of purple basil (*Ocimum basilicum* L. cv. Red Rubin’) were used as the plant material.

Seeds were surface sterilized by being immersed in 70% ethanol for 1 min, followed by a 20 min treatment with 15% sodium hypochlorite. They were rinsed three times with sterile distilled water to eliminate any residual sterilizing agents. Seeds were grown on solid medium containing Murashige and Skoog (MS) medium (MS salts) [30] and 3% sucrose (pH 5.6–5.8), solidified with 0.7% agar, and incubated at 25 °C in the dark to allow for germination. Seed germination was observed after about 10 days, and the seedlings were kept for 4 weeks to achieve full growth. After one month, young leaf explants of approximately 0.5 cm^2^ were cut and placed onto solid medium containing MS salts, 1.0 mg L^−1^ 2.4-D, 0.5 mg L^−1^ kinetin, and different concentrations of NaCl (0, 15, 30, 60, and 90 mM) and treated with 100 mg L^−1^ ZnO NPs to induce callus formation. Zinc oxide nanoparticles (ZnO NPs; Sigma-Aldrich, St. Louis, MO, USA, product no. 544906, MDL no. MFCD00011300) were used in this study. According to the manufacturer, the ZnO NPs were supplied as a nanopowder with a particle size of ≤50 nm and a specific surface area of 10–25 m^2^/g. The ZnO NP suspensions were prepared in distilled water, autoclaved at 120 °C for 20 min, and then sonicated for 30 min at 40 W to inhibit aggregation before being added to the culture medium. The experiment was conducted with three biological replicates per treatment and control (without ZnO NPs). The leaf explants were placed with their abaxial sides in contact with the medium and then moved into a growth chamber controlled under a 16 h light/8 h dark photoperiod at 25 °C. The cultures were maintained in sealed conditions and regularly monitored for callus development. After six weeks, the callus cells were harvested, and the leftover media were removed with filter paper before being weighed for fresh weight analysis. For dry weight measurement, the callus samples were dried at 70 °C for 48 h.

### 4.1. Experimental Design and Treatments

A factorial design was used with salinity (NaCl) and ZnO NP treatments. Each treatment consisted of *n* = 3 independent vessels, each containing five callus pieces. Leaf explants (0.5 cm) of *Ocimum basilicum* L. (purple basil) were cultured on Murashige and Skoog (MS) medium supplemented with 1 mg L^−1^ 2,4-D and 0.5 mg L^−1^ kinetin. The media were prepared with different NaCl concentrations (0, 15, 30, 60, and 90 mM) in the presence or absence of ZnO nanoparticles (100 mg L^−1^) (Table 2). Callus induction was carried out under controlled in vitro conditions over two consecutive culture periods of three weeks each.

### 4.2. Growth Measurements

At harvest, callus fresh weight (FW) was recorded. Dry weight (DW) was obtained after drying samples at 40 °C until constant weight. Growth index (GI) was calculated as GI = (final FW − initial FW)/initial FW.

### 4.3. Determination of Soluble Protein Content

Protein concentration was determined according to the Bradford assay [31]. An appropriate volume of the supernatant was reacted with Bradford reagent containing Coomassie Brilliant Blue G-250. Following a 10 min incubation at room temperature to allow for color development, the absorbance was measured at 595 nm using a spectrophotometer. Protein content was quantified using a standard calibration curve prepared with bovine serum albumin (BSA).

### 4.4. Determination of Oxidative Stress Markers

#### 4.4.1. Hydrogen Peroxide (H_2_O_2_)

H_2_O_2_ content was determined using the potassium iodide (KI) spectrophotometric assay [32]. The absorbance was recorded at 390 nm using a spectrophotometer. H_2_O_2_ concentrations were quantified against a standard calibration curve and expressed as μmol g^−1^ fresh weight (FW).

#### 4.4.2. Lipid Peroxidation (MDA)

Lipid peroxidation was estimated by measuring malondialdehyde (MDA) content using a modified thiobarbituric acid reactive substances (TBARS) assay [33]. The absorbance of the supernatant was recorded at 532 nm, and non-specific turbidity was corrected by subtracting the absorbance at 600 nm. MDA concentrations were calculated using an extinction coefficient of 155 mM^−1^ cm^−1^ and expressed as nmol g^−1^ FW, according to the following equation:

MDA (nmol g^−1^ FW) = [(A_532_ − A_600_)/155,000] × 10^6^ × (V/W)

### 4.5. Antioxidant Enzyme Assays

For enzyme extraction, 0.2 g of callus tissue was thoroughly ground in liquid nitrogen and homogenized in 2 mL of extraction buffer consisting of 0.1 M potassium phosphate KH_2_PO_4_, pH 6.75, 1% (*w*/*v*) polyvinylpyrrolidone (PVP), and 1 mM ethylenediaminetetraacetic acid (EDTA). The homogenate was then centrifuged at 15,000× *g* for 15 min at 4 °C. The resulting supernatant was carefully collected and utilized as the enzyme source for subsequent assays. All extraction and assay procedures were performed at 4 °C to maintain enzyme stability.

Superoxide dismutase activity was determined by its ability to inhibit the photochemical reduction of nitroblue tetrazolium (NBT) [34,35]. Catalase activity was quantified by monitoring the decomposition of H_2_O_2_ at 240 nm [36]. Ascorbate peroxidase activity was assayed by following the rate of ascorbate oxidation at 290 nm [37]. Peroxidase activity was determined using guaiacol as a substrate by measuring the increase in absorbance at 470 nm due to tetraguaiacol formation [38]. All enzyme activities were normalized to the soluble protein content and expressed as U mg^−1^ protein.

### 4.6. GC-MS Metabolomics

#### 4.6.1. Sample Preparation and Volatile Components Analysis

Each plant sample (1.00 g) was finely powdered and transferred into a 10 mL vial, which was then tightly sealed using a rubber silicone septum cap. Prior to analysis, the fiber was conditioned according to the manufacturer’s instructions. After equilibrium was achieved, the fiber was exposed to the vial headspace for 1 min at room temperature. Upon completion of the extraction step, the fiber was withdrawn into the needle and immediately introduced into the injection port of the GC or GC–MS system for analysis.

#### 4.6.2. SPME Analysis

Volatile compounds were characterized using a gas chromatography–mass spectrometry (GC–MS) system. Analyses were performed on a Shimadzu (Kyoto, Japan) GC-MS-QP 2010a instrument operating in electron ionization (EI) mode and equipped with a CP 5MS capillary column (30 m × 0.25 mm i.d., 0.25 μm film thickness). Helium was employed as the carrier gas at a constant flow rate of 1 mL/min. The oven temperature program was initiated at 40 °C and increased to 240 °C at a rate of 2 °C/min, followed by an isothermal hold at 220 °C for 20 min. Both injector and detector temperatures were maintained at 240 °C.

Compound identification was achieved by comparing the linear retention indices (LRIs) with those of a homologous series of n-alkanes (C8–C24), as well as by matching retention times with authentic reference compounds. Mass spectra were recorded at 70 eV. Relative percentages of the identified constituents were determined through area normalization. Following chromatographic separation, the mass spectrum of each compound was individually acquired and identified using the Shimadzu FFNSC Library database.

### 4.7. Statistical Analysis and Integration

Statistical analyses of physiological and enzymatic parameters were performed using JMP software (version 13, SAS Institute Inc., Cary, NC, USA). Data was subjected to analysis of variance (ANOVA), and mean comparisons were conducted using Tukey’s honestly significant difference (HSD) test at a significance level of *p* ≤ 0.05. Principal component analysis (PCA) was additionally applied to evaluate the relationships among the measured traits and treatment groups.

## 5. Conclusions

In conclusion, an integrated enzymatic and GC-MS metabolomic framework demonstrates that 100 mg L^−1^ ZnO nanoparticles significantly mitigate salinity stress in purple basil callus cultures. Our findings reveal that ZnO NPs operate through a coordinated redox-metabolic reprogramming model, characterized by the preferential stimulation of the ascorbate–glutathione cycle (specifically APX activity) and the stabilization of soluble protein content even under high salinity (90 mM NaCl). By reducing oxidative damage markers (H_2_O_2_ and MDA) and partially restoring salinity-perturbed metabolite pools toward a homeostatic state, ZnO NPs act as potent ameliorative agents. This study provides a controlled in vitro platform for mechanistic studies of nanoparticle-stress interactions and highlights the potential of nano-enabled strategies to enhance plant resilience in salt-affected environments.

## Figures and Tables

**Table 1:** The volatile compounds in purple basil callus under salt stress conditions (%).

Group		R.Time	RI	Component	N1	N2	N3	N4	N5	N6	N7	N8	N9	N10
**Oxygenated** **Monoterpenes**	1	19.892	1219	Fenchyl acetate-endo	1.31	0.8	1.52	2.19	2.04		1.97	1.49	1.92	1.19
	2	22.291	1285	Bornyl acetate	2.43	1.29	2.64	2.99	3.26		0.76	3.21	2.4	1.45
	3	24.551	1349	α-Terpinyl acetate			1.07		1.19				1	
	4	30.932	1521	Terpinyl butyrate									0.89	
**Sesquiterpene** **Hydrocarbons**	5	25.450	1375	α-Copaene				0.7	1.03				0.71	0.96
	6	25.996	1390	β-Elemene	2.07	4.95	2.02	3.2	4.46	5.22	1.97	2.07	4.07	4.31
	7	27.354	1445	Seychellene				12.52	7.75		10.66		9.19	17.72
	8	26.610	1418	α-Santalene			0.83	2.29						
	9	26.922	1419	β-Caryophyllene	7.39	8.36	5.47	11.28	8.86	6.07	9.24	8.97	8.52	10.66
	10	26.610	1406	α-Gurjunene		0.9			1.11		1.31	0.92	1.11	1.47
	11	27.346	1414	α-Cedrene	6.12	5.28	3.8			4.42		16.59		
	12	27.436	1432	α-trans-Bergamotene		3.8	1.77	1.67	2.5	0.81	2.21	1.2	3.3	1.73
	13	28.051	1454	α-Humulene	6.94	4.35		6.95	5.54	3.59	9.34	7.01	6.51	5.86
	14	28.571	1482	α-Amorphene				1.29						
	15	28.099	1452	(E)-β-Farnesene	3.43	4.56	9.63	8.52	6.52	1.74	3.48	6.01	6.01	3.7
	16	28.869	1459	α-Patchoulene	1.19							0.92		
	17	29.740	1505	α-Bulnesene		2.17			1.85				1.23	1.05
	18	28.947	1480	Germacrene D	4.26	5.96	4.35	7.2	6.48	2.52	5.81	5.84	5.03	6.59
	19	27.541	1438	Aromadendrene		1.45			1.7				1.43	
	20	29.050	1479	β-Chamigrene	2.29	0.73		7.59	0.83	0.98	3.06	3.69	2.18	5.25
	21	29.149	1494	α-Selinene	2.06		0.93							
	22	29.140	1487	β-Selinene										1.65
	23	29.140	1492	Valencene		0.89	9.34	14.21	5.09	12.57	3.63	5.75	2.39	3.21
	24	30.124	1512	δ-Cadinene	1.36	2.12	1.26	2.29	1.62		1.23	2.96	2.1	4.05
	25	27.698	1449	α-Himachalene							0.94	1.12	0.96	1.84
	26	29.450	1497	Bicyclogermacrene		1.4			1.35		1.25		1.17	
**Oxygenated Sesquiterpenes**	27	34.216	1656	β-Eudesmol				0.93						
	28	34.442	1641	α-epi-Muurolol		2.75	1.47	1.4	1.15		1.49	3.05	2.01	2.35
	29	39.402	1834	Nootkatone			1.03		0.83					
**Alcohols,** **Ketones,** **Aldehydes,** **Furans**	30	35.117	1688	α-Bisabolol			2.28							
	31	40.395	1884	Cetyl alcohol	3	0.6	1.42		1.45		2.03	1.21	1.48	1.94
	32	35.736	1680	Myristic alcohol			2.99				5.4			5.9
	33	43.646	1981	Heptadecyl alcohol	2.9		1.34		0.64		1.02			1.3
	34	37.496	1784	Pentadecanol	15.45	2.25	2.84	2.17	3.91	4.16	3.7	2.37	2.65	4.9
	35	26.440	1403	Eugenol <methyl->	3.68	18.12	13.35		13.43	0.86	11.7		8.55	
	36	43.651	2016	Civetone						1.63				
	37	30.730	1516	Tridecylaldehyde	1.25									
	38	36.741	1749	α-Sinensal			1.47	1.32						
	39	36.784	1754	α-Sinesal						0.99				
**Alkanes,** **Alkenes, Alkynes,** **Arenes**	40	26.207	1400	Tetradecane				0.51		0.86				
	41	29.320	1500	Pentadecane	3.75	0.9				0.75				
	42	32.525	1600	Hexadecane	1.8	1.3	1.63	1.29	1.33	5.12	1.22	2.05		1.35
	43	35.440	1700	Heptadecane	6.93	1.72		0.75		2.47	0.93	1.39		
	44	38.219	1800	Octadecane	0.87	0.79	0.89	0.59		2.44		1.34		
	45	43.391	2001	Eicosane			1.14			1.14				
	46	52.513	2400	Tetracosane						1.15	1.2	1.63	1.51	
	47	39.431	1836	Neophytadiene								1.78		
**Ethers,** **Carboxylic acids,** **Esters**	48	35.744	1682	Undec-10-enoate buty-				1.6	2.16			2.51	2.11	
	49	43.288	1996	Palmitate <ethyl->	16.62	20.55	16.33	3.92	11.94	33.33	12.01	13.76	19.1	6.85
	50	41.576	1925	Palmitate <methyl->		1.13		0.63		3.07		1.17	1.28	0.73
	51	48.032	2198	Stearate <ethyl->		0.88	0.79			1.15				
	52	38.961	1826	Myristate <isopropyl->						1.13				
	53	28.245	1469	γ-Decalactone	0.76									
	54	39.474	1841	Phytone						1.82				
	55	29.929	1502	Phenylacetate methyl-, para-ter>	1.09									
	56	32.860	1610	Lauryl acetate	1.05									
	57	37.490	1781	Caprylate <octyl->			6.4							

**Table 2:** Code assignments for experimental treatment groups.

Numbers	Treatments
N1 = C	Control
N2 = 15	15 mM NaCl
N3 = 30	30 mM NaCl
N4 = 60	60 mM NaCl
N5 = 90	90 mM NaCl
N6 = C + ZNp	Control + ZnO NPs
N7 = 15 + ZNp	15 mM NaCl + ZnO NPs
N8 = 30 + ZNp	30 mM NaCl + ZnO NPs
N9 = 60 + ZNp	60 mM NaCl + ZnO NPs
N10 = 90 + ZNp	90 mM NaCl + ZnO NPs

## Data Availability

The data presented in this study are available upon request from the corresponding author.

## Abbreviations

The following abbreviations are used in this manuscript:

ZnO NPs	Zinc oxide nanoparticles
H_2_O_2_	Hydrogen peroxide
MDA	Malondialdehyde
ROS	Reactive oxygen species
MS	Murashige and Skoog (MS) medium
°C	Celsius degree
DW	Dry weight
FW	Fresh weight
U	Enzyme unit
